# 171 Decline in adherence to Physical Activity Guidelines among the Dutch population

**DOI:** 10.1093/eurpub/ckae114.054

**Published:** 2024-09-26

**Authors:** Tessa Schurink, Saskia van den Berg

**Affiliations:** National Institute for Public Health and the Environment (RIVM), Bilthoven, Netherlands; Centre for Lifecourse, Lifestyle and Health, National Institute for Public Health and the Environment, the Netherlands; National Institute for Public Health and the Environment (RIVM), Bilthoven, Netherlands; Centre for Lifecourse, Lifestyle and Health, National Institute for Public Health and the Environment, the Netherlands

## Abstract

**Purpose:**

In the Netherlands, the number of people who meet the Physical Activity Guidelines in 2022 has dropped dramatically to a level lower than in 2019. In order to get more insight in this decline, we compared PA behavior in 2022 (after the coronavirus pandemic) with 2019 (before the pandemic).

**Methods:**

For this study the 2019 and 2022 data collection of the Health Survey/Lifestyle Monitor (Statistics Netherlands (CBS) in collaboration with National Institute for Public Health and the Environment (RIVM)) was used. This is an annual survey including a random sample of the Dutch population (N ± 10,000). Physical activity was measured with the Short Questionnaire to Assess Health Enhancing Physical Activity (SQUASH). Specifically, time spend on walking and biking to work/school and in leisure time, playing sports, household activities, activities at work or school, gardening and, for children, playing outside and physical education was asked.

**Results:**

In 2022, 44% of the Dutch population 4 years and older was sufficiently physically active compared to 49% in 2019. The decline was highest among young adults aged 18–29 (9.2 percent point), adolescents aged 12–17 (7.5 percent point), people who do paid work (6.4 percent point) and people educated to a higher level (7.5 percent point). All of these groups cycled less in their leisure time (-27, -51, -25 and -22 minutes per week, respectively), although for young adults this difference was not statistically significant. Adolescents also cycled less time to school (-48 minutes per week), but the mean number of days they cycled to school remained the same (4 days a week). In addition, young adults walked less, particularly in their leisure time (-39 minutes per week).

**Conclusions:**

The decline in adherence to the Physical Activity Guidelines is mainly related to a decline in cycling time and, for young adults, a decline in walking time. Especially among adolescents the decrease in the number of minutes cycling between 2019 and 2022 is striking. However, in both 2019 and 2022, they cycled on average 4 days a week to school. A possible explanation for this is the increased use of electric bicycles.

